# Supplementary material for: Modeling glioblastoma heterogeneity as a dynamic network of cell states
Source: Mol Syst Biol. 2021 Sep 16;17(9):e10105. doi: 10.15252/msb.202010105 (PMC8444284; doi:10.15252/msb.202010105)
Supplement: Supplementary file 6 — Source Data for Figure 5 [file MSB-17-e10105-s004.zip › Figure5A_sourcedata/GSEA_3071/hallmarks_stateII.GseaPreranked.1623742536692/HALLMARK_COMPLEMENT.html]

Details for gene set HALLMARK\_COMPLEMENT[GSEA]

|  || Dataset | state23071 |
| Phenotype | NoPhenotypeAvailable |
| Upregulated in class | na\_neg |
| GeneSet | HALLMARK\_COMPLEMENT |
| Enrichment Score (ES) | -0.21040557 |
| Normalized Enrichment Score (NES) | -1.0264091 |
| Nominal p-value | 0.4245614 |
| FDR q-value | 0.7423876 |
| FWER p-Value | 1.0 |
Table: GSEA Results Summary

  

Fig 1: Enrichment plot: HALLMARK\_COMPLEMENT      
 Profile of the Running ES Score & Positions of GeneSet Members on the Rank Ordered List

  

| PROBE | GENE SYMBOL | GENE\_TITLE | RANK IN GENE LIST | RANK METRIC SCORE | RUNNING ES | CORE ENRICHMENT || 1 | F3 |  |  | 13 | 1.555 | 0.0465 | No |
| 2 | PIM1 |  |  | 184 | 0.896 | 0.0331 | No |
| 3 | PLAUR |  |  | 185 | 0.893 | 0.0617 | No |
| 4 | COL4A2 |  |  | 264 | 0.795 | 0.0679 | No |
| 5 | DUSP5 |  |  | 274 | 0.781 | 0.0906 | No |
| 6 | SERPINE1 |  |  | 299 | 0.756 | 0.1089 | No |
| 7 | DGKH |  |  | 301 | 0.754 | 0.1327 | No |
| 8 | CSRP1 |  |  | 518 | 0.619 | 0.0991 | No |
| 9 | CTSC |  |  | 833 | 0.489 | 0.0371 | No |
| 10 | ME1 |  |  | 835 | 0.489 | 0.0525 | No |
| 11 | LRP1 |  |  | 856 | 0.483 | 0.0630 | No |
| 12 | DOCK10 |  |  | 877 | 0.477 | 0.0734 | No |
| 13 | GZMB |  |  | 948 | 0.459 | 0.0707 | No |
| 14 | SH2B3 |  |  | 956 | 0.458 | 0.0836 | No |
| 15 | MAFF |  |  | 986 | 0.447 | 0.0908 | No |
| 16 | PLSCR1 |  |  | 992 | 0.446 | 0.1038 | No |
| 17 | ZFPM2 |  |  | 1023 | 0.437 | 0.1104 | No |
| 18 | TNFAIP3 |  |  | 1070 | 0.424 | 0.1126 | No |
| 19 | IRF1 |  |  | 1173 | 0.403 | 0.1002 | No |
| 20 | USP14 |  |  | 1191 | 0.398 | 0.1087 | No |
| 21 | GPD2 |  |  | 1202 | 0.397 | 0.1190 | No |
| 22 | HSPA1A |  |  | 1409 | 0.356 | 0.0794 | No |
| 23 | EHD1 |  |  | 1413 | 0.354 | 0.0900 | No |
| 24 | RHOG |  |  | 1448 | 0.347 | 0.0927 | No |
| 25 | PFN1 |  |  | 1488 | 0.342 | 0.0940 | No |
| 26 | CD55 |  |  | 1551 | 0.331 | 0.0892 | No |
| 27 | GNAI3 |  |  | 1811 | 0.298 | 0.0347 | No |
| 28 | CASP1 |  |  | 1994 | 0.273 | -0.0016 | No |
| 29 | ANXA5 |  |  | 2187 | 0.253 | -0.0410 | No |
| 30 | DOCK4 |  |  | 2230 | -0.251 | -0.0433 | No |
| 31 | MMP14 |  |  | 2295 | -0.258 | -0.0509 | No |
| 32 | STX4 |  |  | 2374 | -0.267 | -0.0616 | No |
| 33 | PPP2CB |  |  | 2394 | -0.270 | -0.0577 | No |
| 34 | C1R |  |  | 2499 | -0.285 | -0.0743 | No |
| 35 | LAMP2 |  |  | 2724 | -0.321 | -0.1194 | No |
| 36 | CTSO |  |  | 2744 | -0.325 | -0.1137 | No |
| 37 | CBLB |  |  | 2826 | -0.341 | -0.1229 | No |
| 38 | IRF2 |  |  | 2830 | -0.341 | -0.1127 | No |
| 39 | PIK3CA |  |  | 2831 | -0.341 | -0.1018 | No |
| 40 | S100A13 |  |  | 2858 | -0.346 | -0.0972 | No |
| 41 | CALM1 |  |  | 2991 | -0.370 | -0.1180 | No |
| 42 | CTSD |  |  | 3227 | -0.435 | -0.1622 | No |
| 43 | FYN |  |  | 3262 | -0.444 | -0.1564 | No |
| 44 | HSPA5 |  |  | 3335 | -0.468 | -0.1592 | No |
| 45 | GNG2 |  |  | 3543 | -0.567 | -0.1923 | Yes |
| 46 | GNB4 |  |  | 3587 | -0.598 | -0.1838 | Yes |
| 47 | ZEB1 |  |  | 3589 | -0.598 | -0.1649 | Yes |
| 48 | TIMP2 |  |  | 3705 | -0.691 | -0.1712 | Yes |
| 49 | PLAT |  |  | 3766 | -0.752 | -0.1620 | Yes |
| 50 | PRCP |  |  | 3775 | -0.766 | -0.1395 | Yes |
| 51 | GNAI2 |  |  | 3845 | -0.835 | -0.1298 | Yes |
| 52 | DUSP6 |  |  | 3899 | -0.946 | -0.1127 | Yes |
| 53 | CDH13 |  |  | 3908 | -0.963 | -0.0839 | Yes |
| 54 | PDP1 |  |  | 3954 | -1.075 | -0.0606 | Yes |
| 55 | PDGFB |  |  | 3959 | -1.084 | -0.0269 | Yes |
| 56 | CLU |  |  | 4069 | -1.908 | 0.0072 | Yes |
Table: GSEA details [plain text format]

  

Fig 2: HALLMARK\_COMPLEMENT: Random ES distribution      
 Gene set null distribution of ES for **HALLMARK\_COMPLEMENT**
